# Esthetic rehabilitation of crowded maxillary anterior teeth utilizing ceramic veneers: a case report

**DOI:** 10.4076/1757-1626-2-8329

**Published:** 2009-06-29

**Authors:** Süha Türkaslan, Kivanç Utku Ulusoy

**Affiliations:** Department of Prosthodontics, Faculty of Dentistry, Süleyman Demirel University32200, IspartaTurkey

## Abstract

**Introduction:**

In attempting to provide a solution for cases that have been compromised by malpositioned anterior teeth, clinicians traditionally decide orthodontic approach. Nevertheless, in some cases changes in tooth morphology, tooth size, and shape is compulsory for optimal aesthetics. The advent in contemporary aesthetic materials and preparation techniques empowered the clinicians to deliver a promising result with minimal biologic cost. This article presents the clinical considerations that must be addressed when providing a prosthetic restoration for crowded teeth.

**Case presentation:**

A 23 years old Turkish female patient who had malaligned anterior teeth was rehabilitated with ceramic veneers due to her rejection of orthodontic treatment. The paper describes ceramic veneers can be an alternative treatment to orthodontic therapy under variety of preparation forms when patient has anterior malalignment and rejects the orthodontic treatment.

**Conclusion:**

The new smile line was satisfactory for the patient and the esthetics was considered as excellent.

## Introduction

The increasing demand for aesthetic anterior teeth has always encouraged the practitioners to try newly developed materials for more conservative treatment options [1]. The most conservative treatment alternative of crowded anterior teeth is orthodontic approaches. Nevertheless, orthodontic therapy may be rejected by the patient, due to occupational limitations of time and appearance during treatment. The potential for orthodontic relapse has inspired the use of tooth preparation and restorative dentistry to recreate tooth dimensions and proportions commensurate with post-orthodontic results from both an aesthetic and functional clinical outcome, thereby eliminating the potential for relapse [2].

With the introduction of adhesive systems, eliminating the need for full coverage for all-ceramic restorations, more conservative treatment options have been put forward. One of the most minimally invasive techniques is application of laminate veneers [3].

Ceramic veneers not only reduce the destructive approach and minimize the gingival reaction risk of full crowns but also mimic the translucency of natural tooth structure and can provide more promising esthetic results [4]. Several clinical studies have reported the esthetic performance, biocompatibility, and durability of porcelain laminate veneers and their success rate has been clinically evaluated and has shown up to 10 to 15 years [5]. Very few failures were reported due to debonding, microleakage, fracture and caries [6,7]. The incidence of irreparable failure was 7% or less in all of these longitudinal studies. However, the need for intervention without replacement was reported to be as high as 36%, after 10 years [8].

Today, advancement in materials, improvement in fabrication techniques provides different alternatives for the dentists for better success rates. The restoration of the tooth volume utilizing ceramic veneers does not only re-establish the smile but also it allows the biomimetic recovery of the crowns. In the cases which necessitate correction or alteration in tooth shape or position, changes in morphology ceramic veneers display promising esthetic results when clinical procedures carefully carried out [9].

The aim of this current case report is to describe the rehabilitation of malaligned maxillary anterior teeth and the smile line of the patient utilizing lithium disilicate reinforced glass ceramic veneers.

## Case presentation

**Figure 1.**
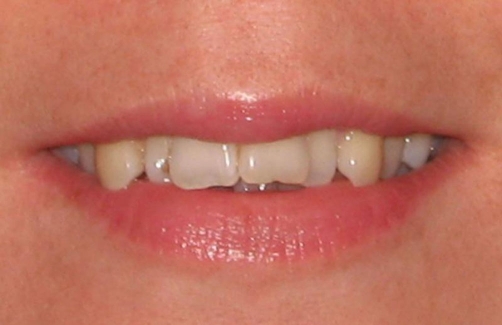
The malaligned maxillary incisors and canines display inharmonious smile.

**Figure 2.**
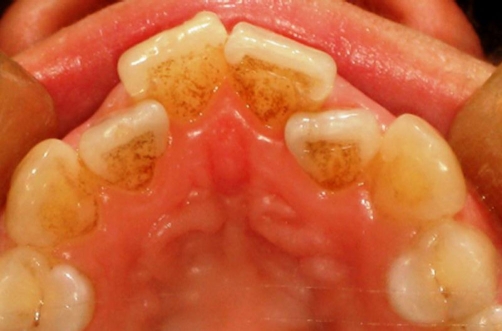
Occlusal view of malalignment in anterior maxillae.

A 23 years old Turkish female patient referred to Süleyman Demirel University Faculty of Dentistry Department of Prosthodontics complaining about the disharmony in maxillary incisors, and canines. The patient’s medical and dental histories were unremarkable. Her oral hygiene performance was satisfactory and flossing was also applied by the patient. Clinical examination revealed the presence of an irregular maxillary incisors and canines (Figure 1). The maxillary dental arch presented two lateral incisors half behind the centrals, rotationally and palatally positioned in the dental arch (Figure 2). Orthodontic therapy was recommended but the patient expressed that she had already experienced several visits in orthodontics clinic but none of the visits helped to persuade her accepting orthodontic therapy.

**Figure 3.**
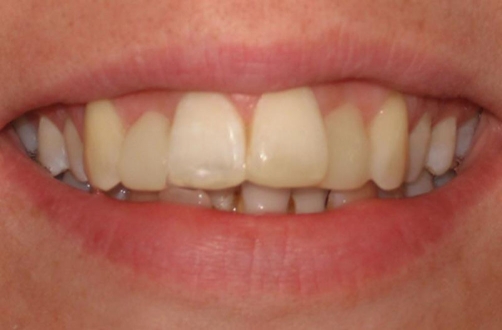
Composite mock-up for predicting the esthetic view.

Restoration of the anterior maxillae utilizing ceramic laminate veneers was decided. Prior to preparation with the composite mock-up technique the esthetic appearance of the patient is predicted. Palatal tooth surfaces and facial gingiva have been isolated with Vaseline (Rosense, Turkey); the facial enamel is spot etched with 37% phosphoric acid etch-gel (Alpha-Etch 37, Dental Technologies, USA) for a few seconds, then rinsed and dried to secure the retention of the future mockup. The incisal part and facial sides of incisors and canines were loaded with A1 shade composite resin material incrementally. Restorative composite is polymerized with LED light curing unit (Freelight Elipar, 3M-ESPE, Germany) for 40 sec. The mock-up is finished and polished with a set of aluminum embedded abrasive discs (Sof-Lex Discs, 3M-ESPE, Germany). The assessment at 2 weeks reveals a harmony between the incisal edge positions and the lower lip. The shape and position of the teeth is discussed and approved by the patient (Figure 3).

**Figure 4.**
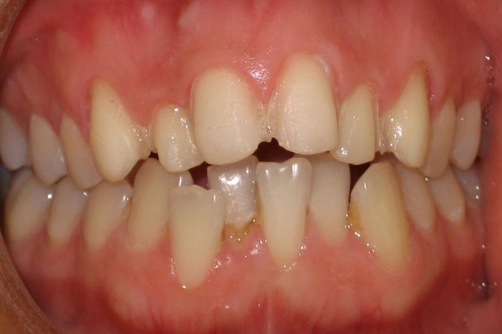
The minimally prepared abutment teeth.

**Figure 5.**
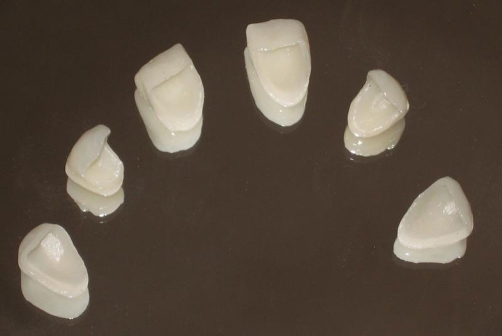
Ceramic veneers ready for the cementation procedure.

During preparation, the facial and palatal surfaces were reduced to 0.5-1.0 mm and the incisal reduction was 1.5 mm. All the incisors and canines were prepared with a chamfered finishing line with rounded internal line angles. The cervical preparation ended at the cemento-enamel junction. Smooth margins were created to prevent stress concentration zones (Figure 4). Once the preparation was completed, impressions were made using polyvinylsiloxane impression material (Elite H-D, Zhermack, Germany). The veneers were waxed up to dies and they were fabricated from lithium disilicate-reinforced glass ceramic material, IPS Empress 2, using the heat press technique according to the manufacturer’s recommendations. After divestment, the veneers were finished and glazed (Figure 5).

The inner surface of ceramic veneers were treated with air-particle abrasion using 50 μm Al_2_O_3_ (Korox, Bego, Germany) with a chairside air-abrasion device (CoJet, 3M-ESPE,Germany) from a distance of 10 mm at a pressure of 250 kPa bar for 10 s. Then each surface treatment was followed by acid etching with 9% hydrofluoric acid (Pulpdent Corporation, USA) prior to silanization. A silane coupling agent (Pulpdent Corporation, USA) was applied to the internal veneer surface for 60 s and air-dried.

During the cementation process each abutment tooth was etched for 15 s using a 37% phosphoric acid etch-gel (Alpha-Etch 37, Dental Technologies, USA). Subsequently, the tooth surface was rinsed thoroughly and air-dried gently. Dentin primer and adhesive were applied according to the manufacturers’ instructions (Clearfil, Kuraray). Following the bonding application a thin layer of light polymerizing composite resin luting cement was applied at the intaglio surface of the veneers, placed onto the prepared teeth and light-polymerized for 40 s (Elipar Free Light, 3M ESPE) from palatal, buccal and incisal sides.

**Figure 6.**
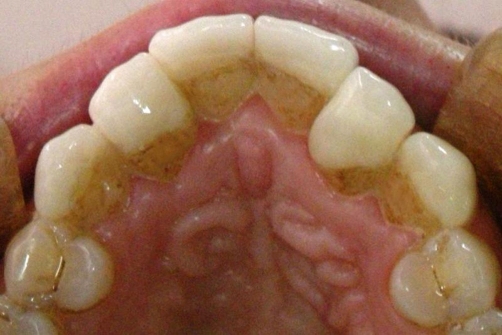
Occlusal view of cemented ceramic veneers.

**Figure 7.**
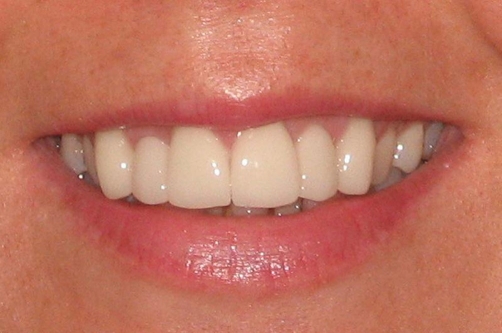
The patient’s new smile with corrected proportions of maxillary incisors and canines.

Excess luting cement was removed and the marginal area was finished and polished with abrasive discs and strips. Restorations were checked to avoid any occlusal interference (Figure 6). The patient was satisfied with her new smile line and excellent view of the anterior teeth (Figure 7) and was recalled in 2 days and encouraged for better dental flossing and also recalled every 6 months for periodical controls. No complication was observed during 3 years clinic service.

## Discussion

Esthetics has become a major component of modern dentistry. Orthodontics can be used to facilitate esthetic dentistry in many ways and it is also the most conservative treatment for remodeling the dental appearance and smile. Most patients can benefit functionally and aesthetically from orthodontic therapy. However, especially adult patients may reject the orthodontic treatment due to occupational time limitation, appearance during treatment or esthetic and psychological concerns. During the past several decades, the orthodontic alternative has been available for the patients [10]. The most conservative and common method for rehabilitating the problem of malalignment without orthodontic therapy is utilizing ceramic laminate veneers. The goals of therapy for the orthodontic and restorative dentistry are similar; how they achieve the results is the only difference [2].

Today, development of modern bleaching techniques, advanced enamel and dentin adhesives, combined with the highly esthetic resin and ceramic materials in esthetic dentistry give chance clinicians to mimic the natural tooth structure. Recently all-ceramic restorations have gained popularity and more frequently preferred in dentistry. Porcelain has been used successfully in dentistry in the form of all-ceramic veneers for the esthetic rehabilitation of teeth. The versatility of veneers allows them to be used with a variety of preparation forms. Ceramic veneers are one of the most conservative and aesthetic techniques that can be applied when restoring the dental arch for improved aesthetics [11]. Their fluorescence is an important physical property in order to mimic the natural tooth. Fluorescence adds to the vitality of a restoration and minimizes the metameric effect between teeth and restorative materials [12]. The use of ceramic facets to solve esthetic and/or functional problems in the anterior section of the dental arch has been shown to be a convincing option. Years of experience with both the technique and the materials employed offer satisfactory, predictable and lasting results [13]. One of the major indications for using PLV is space management [14]. It becomes more of a challenge if the teeth are spaced or not properly aligned on the dental arch, especially in the case of crowded teeth. The dentist is faced problems such as visualizing the aesthetic outcome or tooth preparation when the teeth are not aligned properly on the dental arch [10]. An esthetic examination may also show some other conditions such as severe discoloration or protruding teeth besides crowding that will require additional reduction to achieve esthetic and functional excellence [10].

In this case orthodontic treatment was offered to the patient but the suggestion was rejected. The patient had already dental flossing habit; in addition her oral hygiene was satisfactory.

Another alternative was suggested to patient by using composite mock-up, but due to low fracture strength and not being as durable as ceramics in a dynamic environment like oral cavity, direct or indirect composite veneers could not be planned for this purpose. Therefore, porcelain laminate veneers were optimal solution for the patient. This case presented herein has replicated the treatment outcomes of orthodontic therapy through the use of aesthetic and restorative techniques. The benefits include correction of tooth shapes and dimensions that result in improved tooth proportions with an aesthetically pleasing appearance.

## Conclusion

Ceramic veneers displayed promising results when considering the esthetic and mechanic criteria’s. The new smile of the patient was satisfactory with excellent esthetic appearance.

## Abbreviations

None.
